# Mapping the substrate specificity of the *Plasmodium* M1 and M17 aminopeptidases

**DOI:** 10.1042/BCJ20210172

**Published:** 2021-07-16

**Authors:** Tess R. Malcolm, Karolina W. Swiderska, Brooke K. Hayes, Chaille T. Webb, Marcin Drag, Nyssa Drinkwater, Sheena McGowan

**Affiliations:** 1 Biomedicine Discovery Institute and Department of Microbiology, Monash University, Clayton 3800, Australia; 2 Department of Chemical Biology and Bioimaging, Wroclaw University of Science and Technology, Wyb. Wyspianskiego 27, 50-370 Wroclaw, Poland

**Keywords:** alanyl-aminopeptidase, leucine aminopeptidase, metallo-aminopeptidase, *Plasmodium*, substrate specificity

## Abstract

During malarial infection, *Plasmodium* parasites digest human hemoglobin to obtain free amino acids for protein production and maintenance of osmotic pressure. The *Plasmodium* M1 and M17 aminopeptidases are both postulated to have an essential role in the terminal stages of the hemoglobin digestion process and are validated drug targets for the design of new dual-target anti-malarial compounds. In this study, we profiled the substrate specificity fingerprints and kinetic behaviors of M1 and M17 aminopeptidases from *Plasmodium falciparum* and *Plasmodium vivax*, and the mouse model species, *Plasmodium berghei*. We found that although the *Plasmodium* M1 aminopeptidases share a largely similar, broad specificity at the P1 position, the *P. falciparum* M1 displays the greatest diversity in specificity and *P. berghei* M1 showing a preference for charged P1 residues. In contrast, the *Plasmodium* M17 aminopeptidases share a highly conserved preference for hydrophobic residues at the P1 position. The aminopeptidases also demonstrated intra-peptide sequence specificity, particularly the M1 aminopeptidases, which showed a definitive preference for peptides with fewer negatively charged intrapeptide residues. Overall, the *P. vivax* and *P. berghei* enzymes had a faster substrate turnover rate than the *P. falciparum* enzymes, which we postulate is due to subtle differences in structural dynamicity. Together, these results build a kinetic profile that allows us to better understand the catalytic nuances of the M1 and M17 aminopeptidases from different *Plasmodium* species.

## Introduction

Malaria is one of the world's most prevalent parasitic diseases, most commonly caused by *Plasmodium falciparum* and *Plasmodium vivax*. During the blood stage of infection, *Plasmodium* parasites employ a cascade of proteases to digest hemoglobin to liberate amino acids for use in protein production and maintenance of osmotic pressure within the red blood cell [1]. The alanyl M1 and leucine M17 metalloaminopeptidases from *P. falciparum*, *Pf*A-M1 and *Pf*A-M17, are postulated to play an essential role in the terminal stages of this hemoglobin digestion pathway [2–5]. Attempts at gene knockouts as well as inhibition studies both *in vitro* and *in vivo* have suggested that these proteases are essential and non-redundant [2,4,6,7]. In our laboratory, *Pf*A-M1 and *Pf*A-M17 are the subject of an ongoing structure-based drug design study aimed at generating new non-artemisinin based anti-malarial agents [8–11]. This program has been successful in developing small molecule dual inhibitors of *Pf*A-M1 and *Pf*A-M17 that also show anti-parasitic activity. As anti-malarial resistance becomes more widespread, a requirement for future antimalarials is cross-species activity to ensure that new drugs are effective against both falciparum and vivax malaria [12]. Our latest generation of compounds meet this criterium and possess potent inhibition of the *Plasmodium vivax* M1 and M17 aminopeptidases (*Pv*-M1 and *Pv*-M17). Therefore, the continued development of this dual-target series is complex and demands detailed knowledge about the structure and specificity of each of these targets.

**Figure 1.**
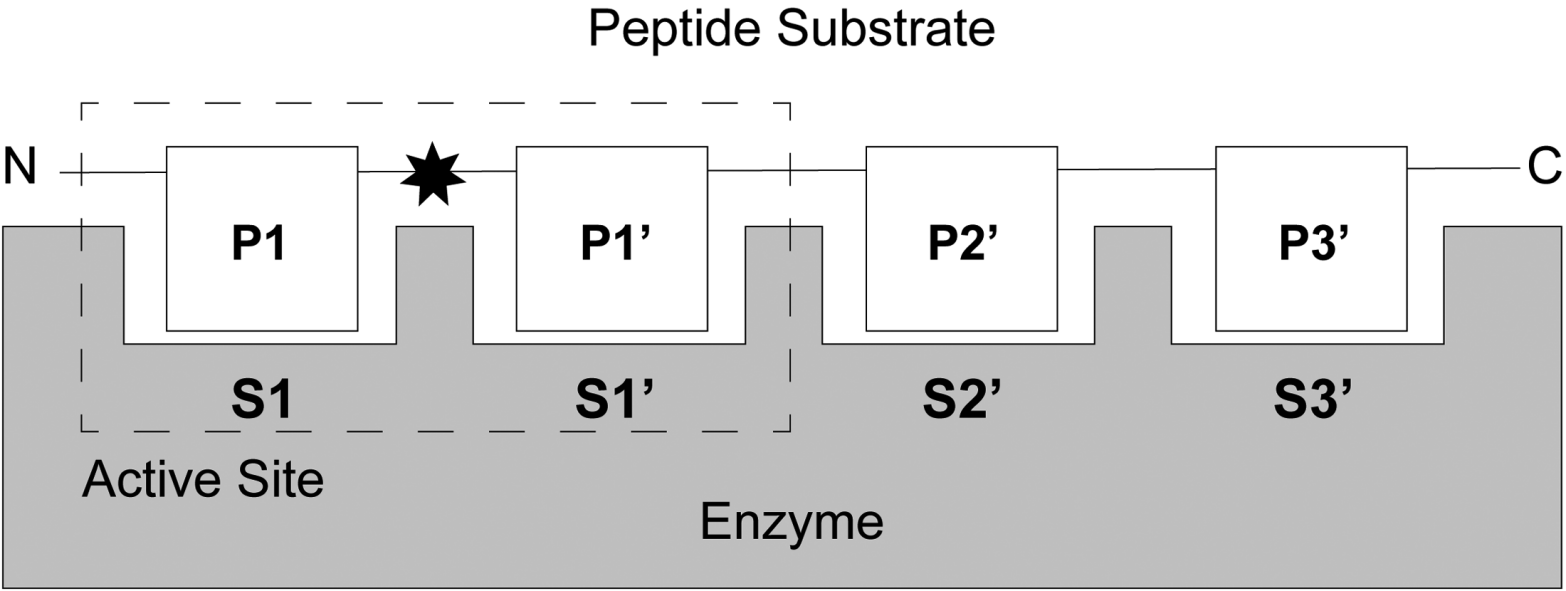
Schematic of aminopeptidase substrate binding site nomenclature. Peptide substrate (white boxes) bind in enzyme (grey) substrate binding sites. The N-terminal residues is denoted as P1, with following residues labeled P1′, P2′ etc. until the C-terminus is reached. Residues are accommodated within their corresponding substrate binding site. The active site is composed of the S1 and S1′ sites, with the hydrolysis reaction occurring between P1 and P1′ denoted by the black star [13].

The extended substrate profiles of the *Plasmodium* alanyl M1 and leucyl M17 aminopeptidases have not been investigated to date. Throughout this study, we have referred to aminopeptidase substrates and substrate binding sites using the standard nomenclature, whereby the N-terminal amino acid is labeled the P1 residue and is accommodated within the S1 substrate binding site (Figure 1) [13]. The following residues are labeled the P1′, P2′ etc. and are accommodated within the S1′, S2′ etc. binding site, until the C-terminus is reached. The S1 and S1′ binding sites constitute the active site, and the hydrolysis reaction occurs between the P1 and P1′ residues (Figure 1) [13]. The P1 substrate specificity of both *Pf*A-M1 and *Pf*A-M17 has been profiled, and both possess a broader substrate specificity range at the P1 position than their names suggest [14]. *Pf*A-M1 has a broad substrate specificity, processing hydrophobic, polar and non-polar residues alike, whereas *Pf*A-M17 has a comparatively narrow substrate specificity profile, with a distinct preference for large hydrophobic residues at the P1 position. Further analysis of the *Pf*A-M1 substrate specificity profile has been carried out, extending into the P1′ and P2′ pockets [15]. *Pf*A-M1 has the highest affinity for di-peptide substrates with large residues at the P1′ position and a comparatively low affinity for di-peptide substrates with small residues at the P1′ position [15]. The P2′ position also favors large, hydrophobic residues [15] and when processing tri-peptides, N-terminal residues are liberated singularly in a processive fashion [16]. Beyond the hydrolysis of tripeptides, there is evidence that suggest both M1 and M17 aminopeptidases are active against long peptide substrates. *Pf*A-M1 has been shown to digest the hemoglobin-derived hexapeptide VDPENF [4] which is not unusual for the enzyme family as the human M1 aminopeptidase ERAP1 has been shown to hydrolyze peptide substrates up to 12 residues long [17]. While *Pf*A-M17 has not been directly shown to hydrolyze long peptide substrates, the M17 aminopeptidase from tomatoes (LAP-A) has been suggested to have activity against a 19-residue precursor to the peptide systemin [18,19].

Here, we describe the substrate profiles for the M1 and M17 aminopeptidases from the malaria causing *P. falciparum* (*Pf*A-M1 and *Pf*A-M17), *P. vivax* (*Pv*-M1 and *Pv*-M17), as well as the parasite used in the murine malaria models, *Plasmodium berghei* (*Pb*-M1 and *Pb*-M17). This study thereby characterizes the substrate profiles of the key *Plasmodium* clinical and animal model species and will inform pre-clinical development of novel inhibitor compound series. The results demonstrate that the activity profiles of the M1 and M17 *Plasmodium* homologs are not only influenced by the N-terminal/P1 residue, but also by substrate length and intra-peptide sequence. This study will underpin our understanding of how substrate characteristics dictate *Plasmodium* M1 and M17 catalytic behavior.

## Materials and methods

### Reagents

Genes encoding *Pb*-M1 (PlasmoDB ID: PBANKA_1410300) and *Pb*-M17 (PlasmoDB ID: PBANKA_1309900) were purchased from GenScript. The synthesized genes were codon optimized for gene expression in *Escherichia coli,* encoded an in-frame C-terminal hexa-histidine tag and were cloned into pET-21d expression vectors. All peptide substrates were purchased from GL Biochem and purified by HPLC to >95% by MS analysis and chemicals purchased from commercial suppliers.

### Production and purification of recombinant aminopeptidases

Recombinant *Pf*A-M1, *Pf*A-M17, *Pv*-M1 and *Pv*-M17 proteins were produced from constructs described previously [11]. Recombinant *Pb*-M1 and *Pb*-M17 were purified using a similar protocol where recombinant protein was expressed in *E. coli* BL21 DE3 cells. Cells were grown in auto-induction media to force overexpression of target proteins before *E. coli* cells were lysed by sonication, centrifuged and the supernatant pooled. Protein was purified via nickel-affinity chromatography, which utilized the encoded C-terminal hexa-histidine tag (HisTrap^TM^ Ni^2+^-NTA column; GE Healthcare Life Sciences), and size-exclusion chromatography (Superdex S20010/300 column; GE Healthcare Life Sciences). All aminopeptidases were stored at −80°C until use; M1 aminopeptidases were stored in 50 mM HEPES pH 8.0, 0.3 M NaCl and 5% glycerol, and M17 aminopeptidases were stored in 50 mM HEPES pH 8.0 and 0.3 M NaCl.

### Enzyme kinetics using fluorogenic substrates

Aminopeptidase enzyme assays were carried out in white 384-well plates (Axygen) in a final volume of 50 µl. Catalytic activity was determined by continual measurement of the liberation of fluorogenic leaving group, 7-amido-4-methyl-coumarin (NHMec) from the commercially available substrate Leucine-7-amido-4-methyl-coumarin (Sigma–Aldrich). Fluorescence was detected using a FLUOStar Optima plate reader (BMG Labtech) with excitation and emission wavelengths of 355 nm and 460 nm, respectively. Assays were carried out in 100 mM Tris pH 8.0 and M17 aminopeptidase assays were supplemented with 1 mM CoCl_2_ unless otherwise stated. Assays screening activity in metal ions included CoCl_2_, MnCl_2_, ZnCl_2_ and MgCl_2_ at varied concentrations between 0 and 1000 µM. Final enzyme concentration for each aminopeptidase was *Pf*A-M1 = 20 nM; *Pf*A-M17 = 125 nM; *Pv*-M1 = 10 nM; *Pv*-M17 = 50 nM; *Pb*-M1 = 20 nM; *Pb*-M17 = 100 nM. For calculation of Michaelis–Menten constants enzyme was kept at a constant concentration and substrate titrated from 0–500 µM. Assays were carried out in biological triplicate, derived from three independent purifications, and data was analyzed using PRISM GraphPad 8 (GraphPad Software, Inc., San Diego, CA). Michaelis–Menten *K*_M_ and *k*_cat_ were calculated using the PRISM GraphPad 8 (GraphPad Software, Inc., San Diego, CA) software and presented as mean ± standard error of the mean (SEM). SEM was used for this data for consistency with previously determined data sets.

The Morrison inhibition constant, *k*_i_^(app)^, of bestatin for *Pv*-M1, *Pv-*M17, *Pb*-M1 and *Pb*-M17 was calculated. Enzyme concentration was kept constant (*Pv*-M1 = 8 nM; *Pv-*M17 = 125 nM; *Pb*-M1 = 20 nM; *Pb*-M17 = 10 nM), substrate concentration was kept constant (*Pv*-M1 = 40 µM; *Pv-*M17 = 10 µM; *Pb*-M1 = 10 µM; *Pb*-M17 = 10 µM), bestatin concentration varied according to potency (*Pv*-M1 = 2 µM–2 nM; *Pv-*M17 = 64 µM–64 nM; *Pb*-M1 = 20 µM–20 nM; *Pb*-M17 = 10 µM–10 nM) and a no compound control included. Aminopeptidase and compound were incubated at 37°C for 10 min prior to addition of substrate. Assays were monitored for 1 h at 37°C with a gain of 800 (FluoStar Optima; BMG LabTech) and were performed in biological triplicate. Morrison *k*_i_ values were calculated by plotting initial velocity versus inhibitor concentration and fitting to the Morrison equation using PRISM GraphPad 8 software (GraphPad Software, Inc., San Diego, CA).

Substrate-profiling at the P1 position was achieved by the use of a fluorogenic substrate library containing 19 natural amino acids and 44 unnatural amino acids [14]. Amino acids were tagged with 7-amino-4-carbamoylmethylcoumarin (ACC) fluorogenic leaving group, which fluoresced upon liberation. Final screening of the library was carried out at 10 µM substrate and 0.50 nM enzyme for M1 aminopeptidase assays, and 500 nM substrate and 6.0 nM enzyme for M17 aminopeptidase assays. Assays were carried out in 50 mM Tris pH 8.0, and M17 aminopeptidase assays were supplemented with 1 mM CoCl_2_. The release of free ACC fluorophore was monitored. For ACC substrates, *K*_M_ values of selected substrates were measured using a range of substrate concentrations unique to each enzyme (*Pv*-M1 = 300 µM–17.6 µM; *Pb*-M1 = 180 µM–10.5 µM; *Pv*-M17, *Pb*-M17 = 10 µM–0.58 µM) and keeping enzyme concentration constant: *Pv*-M1 = 0.672 nM; *Pb*-M1 = 0.336 nM; *Pv*-M17 = *Pb*-M17 = 1.64 nM. Each experiment was repeated at least three times and the average value with standard deviation was calculated. Concentration of DMSO in the assay was <2% (v/v).

### Activity towards peptide substrates

Unlabeled peptide substrates (500 µM) were incubated with enzymes (*Pf*A-M1 = 9.6 µM, *Pf*A-M17 = 17.1 µM, *Pv*-M17 = 16.6 µM, *Pb*-M1 = 9.5 µM, *Pb*-M17 = 18.0 µM) for either 45 min (*Pf*A-M1, *Pf*A*-*M17) or 10 min (*Pv*-M17, *Pb*-M1 and *Pb*-M17) at 37°C in a final reaction volume of 10 µl. *Pv*-M1 cleaved peptides with high efficiency and as such enzyme concentration was decreased to 0.95 µM, peptide concentration reduced to 250 µM and reaction time to 5 min. Assays were carried out in 50 mM Tris pH 8.0 and M17 aminopeptidase assays were supplemented with 1.0 mM CoCl_2_. The reactions were stopped by heating samples at 100°C for 10 min. Samples were analyzed via mass spectrometry using ESI (MicroTOFq; Bruker Daltonics). Data was analyzed using Data Analysis V3.4 Flex Analysis V1.4 (Bruker). The relative percentage of substrate cleaved was calculated by integrating the area under the peptide curve (see Supplementary Figure S3). Experiments were carried out in technical duplicate, derived from a single purification, and percentage peptide cut values were graphed using GraphPad PRISM 8 (GraphPad Software, Inc., San Diego, CA).

## Results

### Characterization of recombinant M1 and M17 aminopeptidases from *P. berghei*

**Figure 2.**
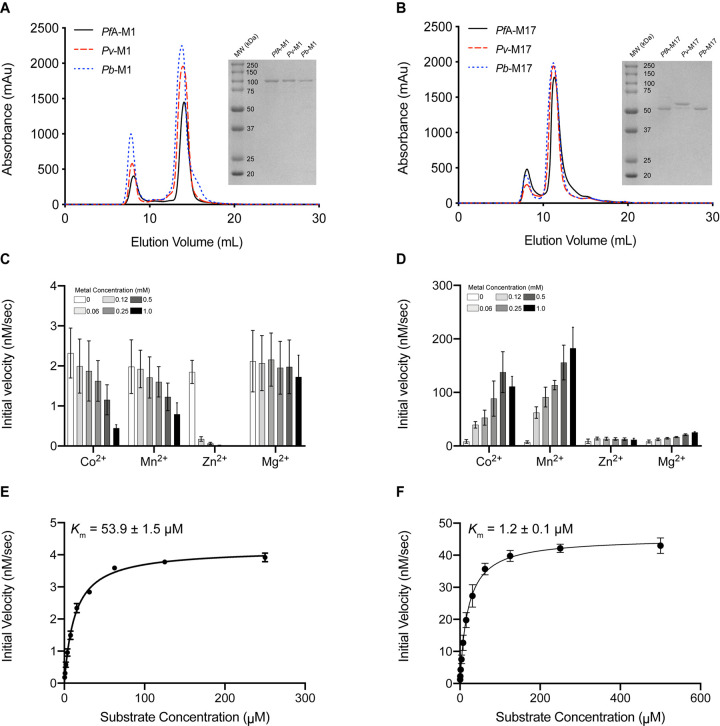
Purification and kinetic characterization of *Plasmodium berghei* M1 and M17 aminopeptidases, *Pb*-M1 and *Pb*-M17. (**A**) Purification profile of *Pf*A-M1 (black), *Pv*-M1 (red dash) and *Pb*-M1 (blue dash) and SDS–PAGE gel (inset) showing correct molecular mass and purity for each homolog. (**B**) Purification profile of *Pf*A-M17 (black), *Pv*-M17 (red dash) and *Pb*-M17 (blue dash) and SDS–PAGE gel (inset) showing correct molecular mass and purity. (**C**) *Pb*-M1 activity profile in presence of increasing concentrations of divalent cations. Activity is highest in the absence of metal ions. (**D**) *Pb*-M17 activity in presence of increasing concentrations of divalent cations. Activity is highest in Co^2+^ and Mn^2+^ and increases as ion concentration increases. (**E**) Michaelis–Menten kinetics of *Pb*-M1. (**F**) Michaelis–Menten kinetics of *Pb*-M17. Assays carried out in biological triplicate and reported as mean ± standard error of the mean (SEM).

The *Pb*-M1 (residues 169–1070) and *Pb*-M17 (residues 109–641) aminopeptidases were successfully expressed using a bacterial expression system. Proteins were purified using an in-frame C-terminal hexa-histidine tag that had previously been optimized for expression and purification of the *Pf*A-M1 and *Pf*A*-*M17 enzymes [3,6]. Both *Pb*-M1 and *Pb*-M17 eluted as a single peak at the same elution volumes as their respective *P. falciparum* and *P. vivax* homologs (Figure 2A,B). All elution profiles exhibited both the main peak containing the desired protein and a void peak at ∼8 ml that is likely aggregated species eluting at the column void volume. The presence of a small tail to the right of the main *Pb*-M17 peak indicates there was a low concentration of smaller *Pb*-M17 species (Figure 2B), although it is not uncommon for M17 family enzymes to display a mix of oligomeric states in the purified sample [20] (Figure 2B). The molecular mass and purity were confirmed using SDS–PAGE and showed that each homolog sample was homogenous and of the expected molecular mass (Figure 2A,B).

We confirmed *Pb*-M1 was active in the absence of supplemental metal ions (Figure 2C), but that *Pb*-M17 required supplementation with Co^2+^ or Mn^2+^ for optimal activity *in vitro* (Figure 2D). This fits with behavior observed from other M1 and M17 aminopeptidases, including *Pf*A- and *Pv*-M1 and M17. The kinetic parameters of *Pb*-M1 were determined in the absence of metal ions. *Pb*-M1 *K*_M_ values for Leu-Mec were ∼5-fold and ∼13-fold lower than *Pf*A-M1 and *Pv*-M1, respectively, and substrate turnover rates (*k*_cat_) were 3.5-fold and 3-fold faster than *Pf*A-M1 and *Pv*-M1, respectively (Table 1). This culminates in *Pb*-M1 processing Leu-Mec with ∼45-fold and ∼13-fold higher efficiency than *Pf*A-M1 and *Pv*-M1, respectively. Although *Pb*-M17 demonstrated optimal activity in 0.5 mM Co^2+^ and 0.25–1.0 mM Mn^2+^ (Figure 2D), kinetic parameters were determined in the presence of 1 mM Co^2+^ to align with previously published experiments [11]. The *P. berghei* homolog was also the most efficient M17 aminopeptidase tested, with *K*_M_ values akin to *Pf*A*-*M17 and *Pv-*M17, albeit slightly lower, but extremely elevated substrate turnover rates. *Pb*-M17 is ∼84-fold more efficient than *Pv-*M17 and ∼440-fold more efficient than *Pf*A*-*M17 at catalyzing Leu-Mec (Table 1).

**Table 1:** Kinetic parameters of PfA-M1, Pv-M1, Pb-M1, PfA-M17, Pv-M17 and Pb-M17 determined using Leu-Mec fluorescent substrate

	*K*_M_ (µM)	*k*_cat_ (s^−1^)	*k*_cat_/*K*_M_ (M^−1^•s^−1^)
*Pf*A-M1	173 ± 0.7	0.12 ± 0.004	6.9 × 10^2^
*Pv*-M1	61.9 ± 8	0.14 ± 0.004	2.3 × 10^3^
*Pb*-M1	13.1 ± 2.6	0.42 ± 0.006	3.1 × 10^4^
*Pf*A*-*M17	40.9 ± 11	0.01 ± 0.0009	2.5 × 10^2^
*Pv-*M17	31.0 ± 17	0.04 ± 0.005	1.3 × 10^3^
*Pb-*M17	19.9 ± 3.9	2.27 ± 0.09	1.1 × 10^5^

Assays carried out in biological triplicate and reported as ± standard error of the mean (SEM).

The sequences of each of the three homologs were aligned and compared. M1 aminopeptidases typically assume a monomeric structure composed of four domains (Supplementary Figure S1: Domain I, blue; Domain II, yellow; Domain III, green; Domain IV, pink), with the catalytic active site contained within Domain II. In keeping with this, Domain II has the highest overall sequence similarity and all residues involved in metal binding (Supplementary Figure S1: marked with cross) and the catalytic machinery (Supplementary Figure S1: boxed residues) are conserved among *Pf*A-M1, *Pv*-M1 and *Pb*-M1. *Pb*-M1 exhibits the conserved GAMEN motif (residues 435–439) and HEYFHX_17_KE zinc binding domain that are characteristic of M1 aminopeptidases. Domain III and Domain IV are the most divergent, with *Pv*-M1 and *Pb*-M1 exhibiting several short stretches of residues within Domain IV that are not observed in *Pf*A-M1 (Supplementary Figure S1).

M17 aminopeptidases adopt a homo-hexameric conformation; each of the six chains are composed of a solvent exposed N-terminal domain (Supplementary Figure S2, blue), a helical linker (Supplementary Figure S2, yellow), and an internal catalytic C-terminal domain (Supplementary Figure S2, green). The N-terminal domains are highly divergent, particularly *Pv-*M17, which features an extended flexible loop that is not observed in either *Pf*A*-*M17 or *Pb*-M17. The catalytic C-terminal domain however is highly conserved, and all metal-binding residues (Supplementary Figure S2, marked with crosses) and catalytic residues (Supplementary Figure S2, crossed residues) are conserved.

### Comparing the substrate specificity at the P1 position across the *Plasmodium* M1 aminopeptidases

**Figure 3.**
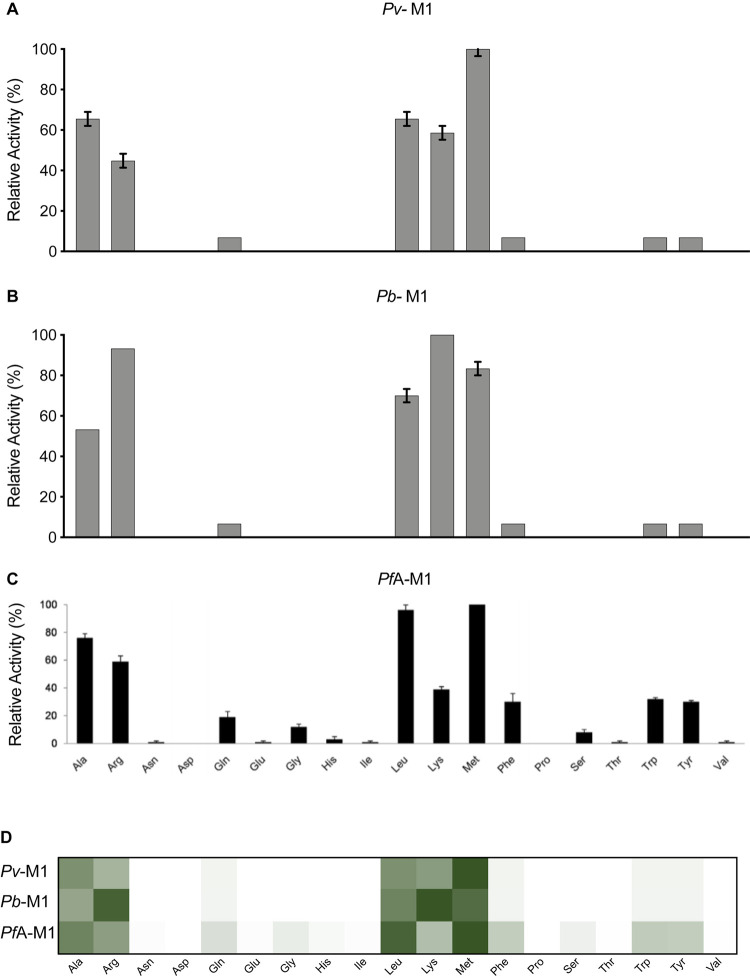
P1 natural amino acid substrate profile of M1 aminopeptidases from *P. falciparum*, *P. vivax* and *P. berghei*. P1 substrate specificity screening of *Pv*-M1 (**A**), *Pb*-M1 (**B**) and *Pf*A-M1 (**C**) [14] with a 19-membered natural amino acid library. The amino acid with highest activity recorded was assigned as 100% activity, and activity against other amino acids is represented as percentage activity. (**D**) Heat map depicting the most favored amino acids as dark green and least favored as white, with all intermediate values depicted as shades of green. Error bars represent *± *standard deviation (SD). Experiments were carried out in biological triplicate.

To profile the P1 substrate specificities of the enzymes, we used the same fluorogenic substrate library that we have used previously to characterize *Pf*A-M1 and *Pf*A*-*M17 [14]. This library contains 19 natural and 44 unnatural amino acids coupled to an ACC leaving group. The only natural amino acid not tested was cysteine, to avoid oxidation issues. To compare enzyme selectivity, the amount of fluorescent end-product generated from each substrate within the experimental time frame was measured. The substrate that produced the highest fluorescence levels within the designated time frame was assigned a value of 100% and used to calculate relative digestion levels of other substrates (Figure 3). The results show that *Pv*-M1 and *Pb*-M1 are both most selective for the same natural P1 amino acids (Ala, Arg, Leu, Lys, Met, Phe, Trp, Tyr, Gln) but their relative activities against each is quite different. *Pv*-M1 had a P1 preference for Met > Ala > Leu before the charged and/or polar residues Lys > Arg >> Gln. (Figure 3A). Interestingly, the preference for hydrophobic residues is reversed in *Pb*-M1 that preferred Lys/Arg before the bulkier hydrophobics Met > Leu and then Ala (Figure 3B). Including previous *Pf*A-M1 data in the comparison (Figure 3C), we can see that the *Pf*A-M1 profile is more similar to that of *Pv*-M1, sharing a preference for the hydrophobic sidechains. *Pf*A-M1 appears to have a slightly broader profile as it shows activity toward smaller residues like Ser, Gly, Val and Thr (Figure 3C). The enzymes were the least active in removal of the aromatic side chains (Phe = Trp = Tyr). The heat map highlights that all three homologs are highly conserved in the residues that are preferentially cleaved, with more variation in substrate specificity at lower activity levels (Figure 3D).

**Figure 4.**
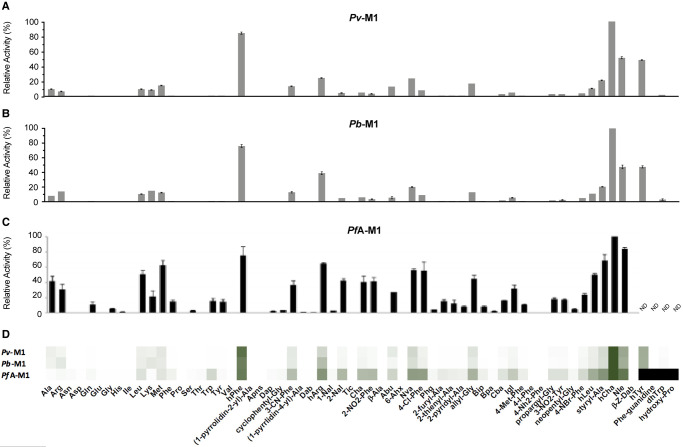
P1 unnatural and natural amino acid substrate profile of M1 aminopeptidases from *P. falciparum*, *P. vivax* and *P. berghei*. P1 substrate specificity screening of *Pv*-M1 (**A**), *Pb*-M1 (**B**) and *Pf*A-M1 (**C**) [14] with the 44-membered unnatural amino acid library. The amino acid with highest activity recorded was assigned as 100% activity, and activity against other amino acids is represented as percentage activity. (**D**) Heat map depicting the most favored amino acids as dark green and least favored as white, with all intermediate values depicted as shades of green. Black boxes in heat map and ND in panel **C** (not determined) represent substrates that were not tested against *Pf*A*-*M1 as part of Poreba et al. (2011) study [14]. Experiments were carried out in biological triplicate. Error bars represent *± *standard deviation (SD).

Each of the M1 aminopeptidases demonstrated elevated activity against the unnatural amino acid substrates compared with their natural counterparts. This high activity against unnatural amino acids is likely due to the comparatively larger size of the unnatural amino acids. *Pv*- and *Pb*-M1 processed the 44-membered unnatural amino acid library with very similar substrate specificity profiles (Figure 4). *Pv*-M1 had highest activity against hCha and hPhe, moderate activity against Nle and hTyr and low activity against hArg, Nva, styryl-Ala, allyl-Gly, 3-CN-Phe, Abu and hLeu. *Pb*-M1 shared this specificity, although had high activity against Arg derivative, hArg, and low activity against Ala derivative, Abu. The *Pb*-M1 preference for Arg over Ala is observed in both natural amino acids and their derivatives. The *Pf*A-M1 profile exhibits some differences to both the *Pv*-M1 and *Pb*-M1 profiles, most notably at low activity levels (Figure 4C). At high activity levels, preferential cleavage of hCha, hArg, hPhe, styryl-Ala and Nle is conserved, while at low activity levels *Pf*A-M1 exhibits a much broader specificity range. *Pf*A-M1 had low activity against substrates that are not hydrolyzed by *Pb*-M1 and *Pv*-M1, including Dap, cyclophentyl-Gly, (1-pyridin-4-yl)-Ala, Dab, 1-Nal, Phg, Bpa and neopentyl-Gly. Comparison of the heat map indicates that while *Pf*A-M1 has unique low-level activity against some unnatural residues, specificity is mostly conserved between the three homologs (Figure 4D).

The kinetic parameters of *Pv*-M1 and *Pb*-M1 were evaluated using the most preferred substrate, hCha (Table 2). *Pv*-M1 and *Pb*-M1 *K*_M_ and substrate turnover values were within 2-fold, although *Pv*-M1 had slightly lower *K*_M_ values and faster turnover rates (Table 2). As a result, *Pv*-M1 had a marginally higher catalytic efficiency than *Pb*-M1 (*Pv*-M1 *k*_cat_/*K*_M_ = 7.6 × 10^5^ M^−1^ s^−1^; *Pb*-M1 *k*_cat_/*K*_M_ = 3.0 × 10^5^ M^−1^ s^−1^). Previously determined data shows that *Pf*A*-*M1 processes the same substrate with 15 to 40-fold less efficiency (*Pf*A-M1 *k*_cat_/*K*_M_ = 1.7 × 10^4^ M^−1^ s^−1^), largely due to a 10 to 14-fold decrease in substrate turnover rate [14].

**Table 2:** Kinetic parameters of PfA-M1, Pv-M1, Pb-M1, PfA-M17, Pv-M17 and Pb-M17 against unnatural amino acid substrate hCha

	*K*_M_ (µM)	*k*_cat_ (s^−1^)	*k*_cat_/*K*_M_ (M^−1^•s^−1^)
*Pf*A-M1	96.3 ± 16.2	1.6 ± 0.2	1.7 × 10^4^
*Pv-*M1	29.6 ± 4.4	22.4 ± 0.9	7.6 × 10^5^
*Pb-*M1	53.9 ± 1.5	16.4 ± 2.9	3.0 × 10^5^
*Pf*A-M17	0.44 ± 0.02	0.21 ± 0.005	4.8 × 10^5^
*Pv-*M17	1.1 ± 0.03	0.4 ± 0.04	3.6 × 10^5^
*Pb*-M17	1.2 ± 0.1	0.3 ± 0.03	2.5 × 10^5^

Assays carried out in biological triplicate and reported as mean *± *standard deviation (SD).

### M1 aminopeptidases demonstrate intra-peptide sequence specificity

**Figure 5.**
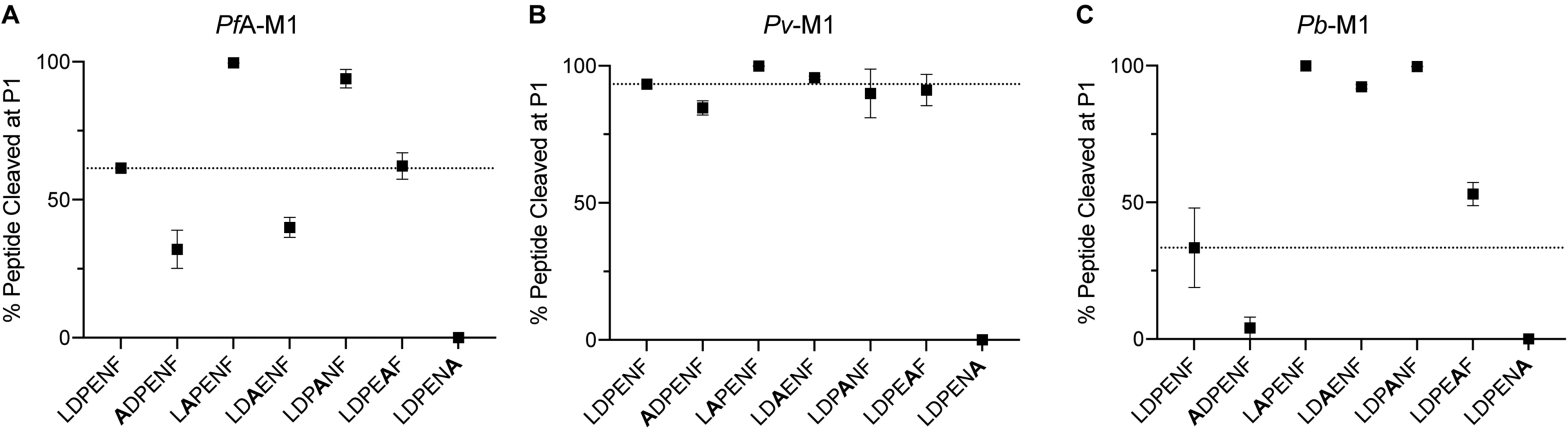
Activity of *Pf*A*-*M1, *Pv-*M1 and *Pb*-M1 against hemoglobin-derived hexapeptide and alanine screen peptides. Activity of *Pf*A*-*M1 (**A**), *Pv-*M1 (**B**) and *Pb*-M1 (**C**) against hemoglobin derived hexapeptide, LDPENF, and alanine screen peptides, ADPENF, LAPENF, LDAENF, LDPANF, LDPEAF and LDPENA. Activity presented as percentage of peptide cleaved at the P1 position. Dashed line represents activity against template peptide LDPENF. Experiments were carried out in technical duplicate. Squares represent duplicate mean and error bars represent duplicate range.

**Figure 6.**
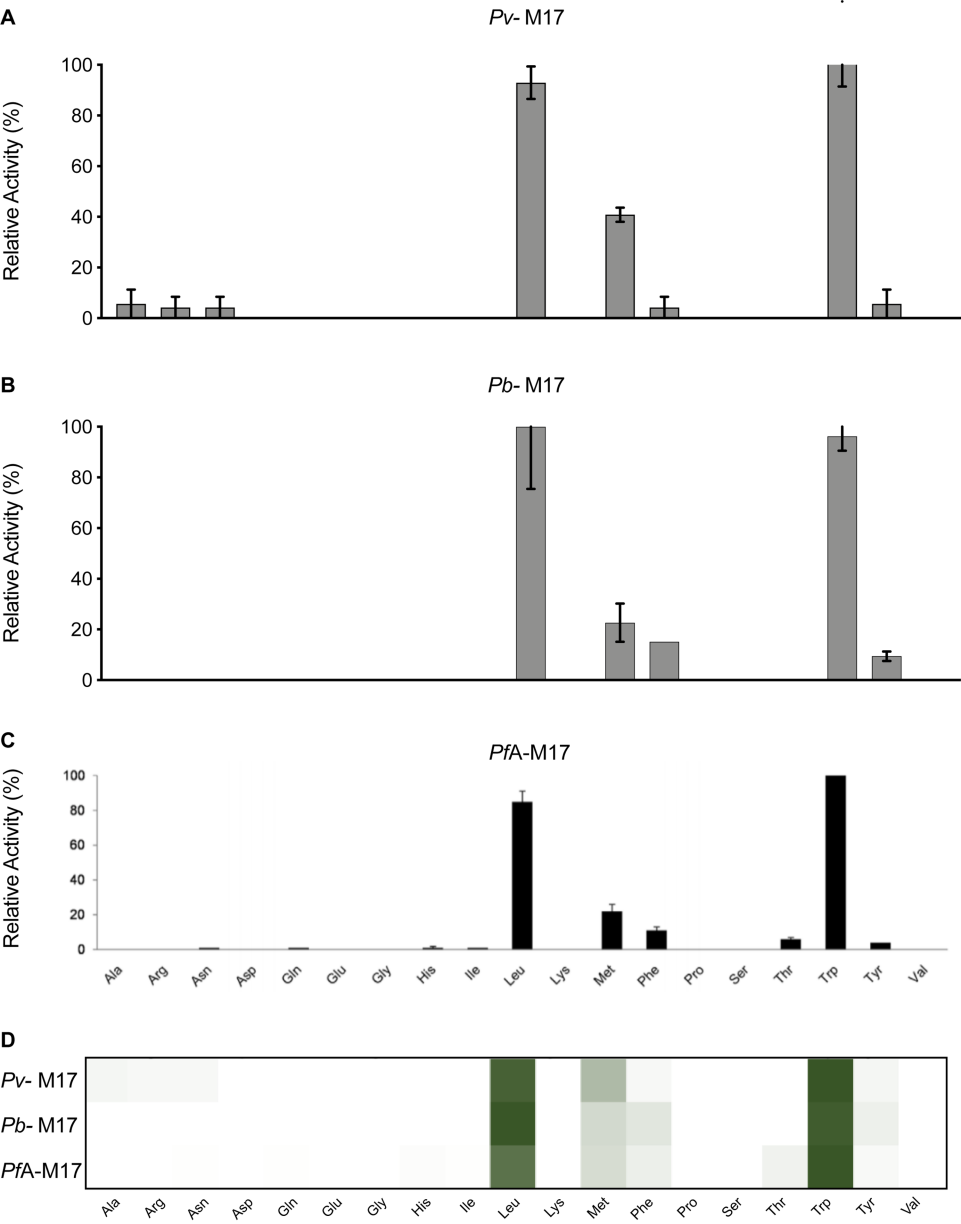
P1 natural amino acid substrate profile of M17 aminopeptidases from *P. falciparum*, *P. vivax* and *P. berghei*. P1 substrate specificity screening of *Pv*-M17 (**A**), *Pb*-M17 (**B**) and *Pf*A-M17 (**C**) [14] with a 19-membered natural amino acid library. The amino acid with highest activity recorded was assigned as 100% activity, and activity against other amino acids is represented as percentage activity. (**D**) Heat map depicting the most favored amino acids as dark green and least favored as white, with all intermediate values depicted as shades of green. Experiments were carried out in biological triplicate. Error bars represent *± *standard deviation (SD).

Previous studies had shown that *PfA*-M1 could degrade hexapeptides derived from hemoglobin (α chain peptide VDPENF and β chain peptide VDPVNF) [4]. We used the naturally occurring α chain peptide (VDPENF) as a template to produce a substrate derivative digestible by both M1 and M17 aminopeptidases. This was achieved by substituting the P1 Val residue, which only the M1 aminopeptidases can process, to Leu (LDPENF). Using mass spectrometry to detect the end point product of digestion, we were able to show that both the *Plasmodium* M1 and M17 enzymes cleaved the P1 Leu from this hexapeptide (Figures 5, 8). We also anticipated that the P1′ Asp residue in the peptide sequence would prevent processive activity, as none of the enzymes could efficiently cleave Asp in the P1 position (Figures 3, 6). This was indeed the case, as DPENF was the only smaller peptide fragment detected from our end point experiments (Supplementary Figure S3).

Allary et al. [16] and Dalal et al. [15] both demonstrated that *Pf*A-M1 has specificity beyond the P1 site. The digestible LDPENF hexapeptide was used as a template in the design of an alanine screen peptide library used to test the intra-peptide specificities of *Pf*A-M1, *Pv*-M1 and *Pb*-M1. Each peptide within the library featured Ala substituted at a different position to further understand what residues are accepted at each position, and which intrapeptide substitutions resulted in substrates that could be hydrolyzed. Peptide cleavage was detected using mass spectrometry; both cleaved and uncleaved peptides were detected and used to calculate the percentage cleavage of each peptide calculated from the total peptide amount detected. Assay times were reduced for *Pv*-M1 and *Pb*-M1 as all peptides were cleaved to almost 100% within the 45 min timeframe, resulting in a loss of specificity information (Supplementary Figure S4).

*Pf*A-M1 showed a preference for LAPENF and LDPANF over LDPENF (Figure 5A). When Ala replaced Asp in the P1′ position, activity increased from 61% to 100%. Similarly, the substitution of Glu for Ala in the P3′ position resulted in increased activity (94%). The peptide product, APENF, produced from the cleavage of LAPENF was not further digested due to the inability of *Pf*A-M1 to cleave peptide with a P1′ Pro. *Pf*A-M1 functioned more efficiently when negatively charged residues were replaced, regardless of their position within the peptide (Figure 5A). Replacement of P1 Leu with Ala resulted in a decrease in activity from 61% to 32%, as *Pf*A-M1 prefered Leu in the P1 position (a result supported by the P1 screen, Figure 3). Substitution of the P2′ Pro residue (LDAENF) similarly resulted in decreased activity (40%), suggesting the rigidity that the Pro residue provides to the peptide may be vital for optimal activity. LDPENA was the only peptide that *Pf*A-M1 did not cleave, which is likely a result of replacing a large aromatic residue with the comparatively small Ala sidechain. *Pv*-M1 was highly efficient at cleaving the hemoglobin-derived peptide and alanine screen peptides. Even with the reduction in enzyme concentration, peptide concentration and incubation time, activity rates were in excess of 80% for each peptide, except for LDPENA, which was not processed at all within the shorter timeframe. *Pv*-M1 demonstrated a slightly lower tolerance for Ala in the P1 position than Leu, with activity rates dropping from 93% to 84%. The substitution of Ala in the P1′ position in place of Asp to produce LAPENF increased activity to 99%, while the activity levels of LDAENF, LDPANF and LDPEAF remained approximately similar to LDPENF. *Pb*-M1 preferentially cleaved both LAPENF and LDPANF over LDPENF. *Pb*-M1 had 99% activity against both LAPENF and LDPANF, compared with the 33% activity against LDPENF, thereby exhibiting a preference for peptides with fewer negatively charged residues. *Pb*-M1 also showed a preference for Ala in the P2′ position in place of Pro (LDAENF), with the substitution increasing activity to 92%. The substitution of Pro for Ala in the P2′ position results in an inherently more flexible peptide, which is advantageous for enzyme activity. *Pb*-M1 showed decreased activity against both ADPENF and LDPENA. *Pb*-M1 catalyzed ADPENF with activity rate of only 4%, while there was no observed cleavage of LDPENA.

### The *Plasmodium* M17 aminopeptidase P1 profile is highly conserved

The P1 substrate specificity of the M17 aminopeptidases was profiled using the same 19-membered natural amino acid and 44-membered unnatural amino acid library used to profile the M1 aminopeptidases. Compared with the M1 homologs, the M17 aminopeptidases had a very limited substrate specificity range. *Pv-*M17 demonstrated a preference for the bulky nonpolar residues Trp, Leu and Met and showed low-level activity (<10%) against Ala, Arg, Asn, Phe and Tyr (Figure 6A). *Pb*-M17 demonstrated a similar preference for the bulky hydrophobic residues, although catalyzed the removal of Leu with the highest efficiency followed by Trp and Met. *Pb*-M17 was less efficient at processing Met than *Pv-*M17 but showed higher activity against aromatic residues Phe and Tyr (Figure 6B). When compared with the previously determined *Pf*A-M17 data, we can see the profiles are very much conserved between homologs (Figure 6C). The heat map indicated a conserved preference for Leu and Trp, followed by Met, and finally Phe and Tyr (Figure 6D). *Pv-*M17 and *Pf*A*-*M17 [14] both had low-level activities against several substrates that was not conserved between homologs.

**Figure 7.**
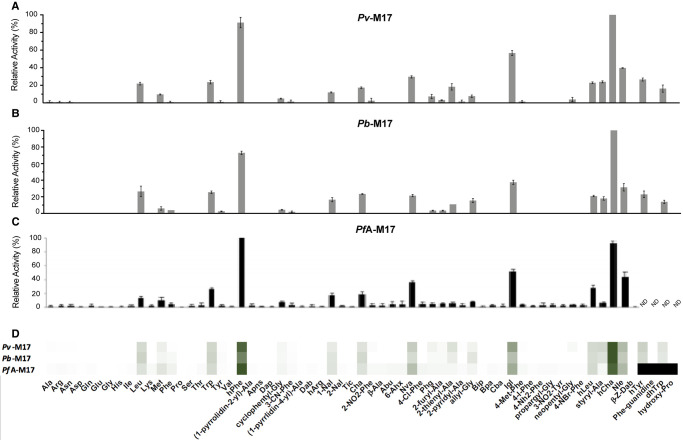
P1 unnatural and natural amino acid substrate profile of M17 aminopeptidases from *P. falciparum*, *P. vivax* and *P. berghei*. P1 substrate specificity screening of *Pv*-M17 (**A**), *Pb*-M17 (**B**) and *Pf*A-M17 (**C**) [14] with the 44-membered unnatural amino acid library. The amino acid with highest activity recorded was assigned as 100% activity, and activity against other amino acids is represented as percentage activity. (**D**) Heat map depicting the most favored amino acids as dark green and least favored as white, with all intermediate values depicted as shades of green. Black boxes in heat map and ND in panel **C** (not determined) are substrates that were not tested against *Pf*A*-*M17 as part of Poreba et al. (2011) [14] study. Experiments were carried out in biological triplicate. Error bars represent *± *standard deviation (SD).

The M17 aminopeptidases cleaved the unnatural amino acids with much higher efficiency than the natural amino acids and activity profiles were again largely conserved across the three homologs. Similar to the M1 aminopeptidases, the preference for unnatural amino acids over their natural counterparts is observed and is likely due to the preference of M17 aminopeptidases for bulkier substituents. *Pv-*M17 and *Pb*-M17 demonstrated similar specificity profiles; both aminopeptidases preferentially cleaved hCha > hPhe > Igl > Nle with highest efficiency, and cleaved hCha with 4-fold higher efficiency than the best natural amino acid (Figure 7A,B). *Pv-*M17 then preferentially cleaved Nva > hTyr > styryl-Ala > hLeu, while *Pb*-M17 then preferentially cleaved Cha > hTyr > Nva > hLeu. *Pv-*M17 had higher activity against Val and Ala derivatives Nva and styryl-Ala (Figure 7A), while *Pb*-M17 had a unique preference for Ala derivative Cha (Figure 7B). When comparing to the previously determined *Pf*A*-*M17 data [14], the strong preferences observed for hPhe, hCha, Igl and Nle are conserved across the three homologs. *Pf*A*-*M17 also showed higher activity levels against the unnatural amino acids compared with the natural amino acids, cleaving the most preferred unnatural substrate (hPhe) with 4-fold higher efficiency than the best natural substrate (Trp). The heat map indicates that the substrates processed with high efficiency were conserved across the three homologs, whereas the substrates that are processed with low efficiency are more unique to each homolog (Figure 7D).

For each of the homologs, we determined the kinetic parameters for the unnatural amino acid substrate, hCha (Table 2). *Pv-*M17 and *Pb*-M17 had remarkably similar kinetic parameters, with *K*_M_ values of 1.1 µM and 1.2 µM, respectively, and turnover rates of 0.4 and 0.3 s^−1^, respectively. Together, these result in catalytic efficiencies of 3.6 × 10^5^ and 2.5 × 10^5^ M^−1^ s^−1^, respectively. The previously determined *Pf*A*-*M17 results illustrate a slightly higher catalytic efficiency (4.8 × 10^5^ M^−1^ s^−1^) for hCha than *Pv-*M17 and *Pb-*M17, which was largely due to the lower *K*_M_ values of *Pf*A*-*M17 for hCha; *K*_M_ = 0.44 µM (Table 2) [14].

### Intrapeptide sequence specificity is highly conserved between M17 homologs, and integral to catalytic efficiency

**Figure 8.**
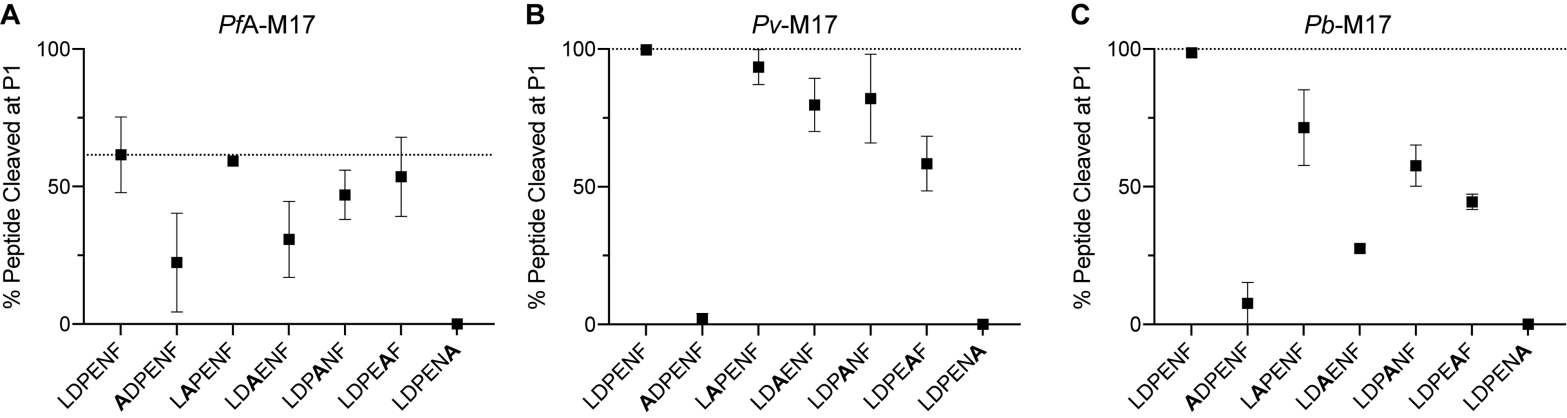
Activity of *Pf*A*-*M17, *Pv-*M17 and *Pb*-M17 against hemoglobin-derived hexapeptide and alanine screen peptides. Activity of *Pf*A*-*M17 (**A**), *Pv-*M17 (**B**) and *Pb*-M17 (**C**) against hemoglobin derived hexapeptide, LDPENF, and alanine screen peptides, ADPENF, LAPENF, LDAENF, LDPANF, LDPEAF and LDPENA. Activity presented as percentage of peptide cleaved at the P1 position. Dashed line represents activity against template peptide LDPENF. Experiments were carried out in technical duplicate. Squares represent duplicate mean and error bars represent duplicate range.

We used the same Ala peptide library screen to assess intrapeptide specificity within the M17 aminopeptidases. Similar to the M1s, *Pf*A*-*M17 digested peptides at a much slower rate compared with *Pv-*M17 and *Pb*-M17. To account for this, *Pf*A*-*M17 experiments were carried out for a total of 45 min, whereas *Pv-*M17 and *Pb*-M17 experiments were carried out for 10 min (*Pv-*M17 and *Pb*-M17 digested all peptides to almost 100% in the 45 min time frame, Supplementary Figure S5). All experiments were carried out in technical duplicate; however, we did observe notable differences between the two *Pf*A*-*M17 replicates. Despite this, the two replicates had a conserved order of preference for the peptides tested. (LAPENF/LDPENF/LDPEAF > LDPANF > LDAENF > ADPENF > LDPENA) (Figure 8A). *Pf*A*-*M17 cleaved the hemoglobin-derived hexapeptide with 62% efficiency and did not show enhanced activity for any other peptides. LAPENF, LDPANF and LDPEAF were cleaved with similar efficiency to LDPENF, at rates of 59%, 47% and 53%, respectively. The only substitutions that resulted in a change in activity were at the P1, P2′ and P5′ positions (Figure 8A). The substitution of Leu for Ala in the P1 position was clearly unfavorable, with activity rates dropping to 22% due to the preference for Leu in the P1 position. Replacing the P2′ Pro with Ala reduced activity to 31%, likely due to the increase in peptide flexibility that an Ala in that position provides. The substitution of Phe for Ala in the P5′ position had the most dramatic effect on substrate specificity, reducing activity to <1%. Like *Pf*A-M1, *Pf*A*-*M17 appears to require a bulky aromatic residue on the C-terminus to successfully cleave the peptide substrate. *Pv-*M17 cleaved LDPENF with the highest efficiency compared with the other peptides, and any substitutions to the sequence resulted in no change in activity or in some cases, a decrease in activity (Figure 8B). LAPENF and LDPANF were within a similar activity rate range to LDPENF, indicating that P1′ and P3′ positions tolerate Ala the same as, or slightly less than, Asp and Glu, respectively. *Pv-*M17 had a unique tolerance for Ala in the P2′ position in place of Pro, with activity rates of 80%. Substitution of Asn to Ala in the P4′ position reduced activity by almost half from 99% to 58%, while substitution to Ala in the P1 and P5′ essentially ablated all activity. *Pb*-M17 also digested hemoglobin-derived peptide (LDPENF) with the highest efficiency with 98% cleaved, however showed a decrease in activity against LAPENF and LDPANF that was unique amongst the three homologs (Figure 8C). LAPENF was digested to 71%, and LDPANF digested to 57%, compared with the 98% cleavage rate before substitution. Substitution of Pro for Ala at the P2′ position (LDAENF) and Asn for Ala at the P4′ position (LDPEAF) reduced activity to 24% and 44%, respectively, and substitution of Leu for Ala in the P1 position (ADPENF) and Phe for Ala in the P5′ position (LDPENA) further reduced activity to 7% and 0%, respectively.

### Bestatin can potently inhibit both M1 and M17 aminopeptidases

The M1 and M17 aminopeptidase families demonstrate unique substrate specificity profiles, with M1 aminopeptidases favoring charged and hydrophobic residues in the P1 position and M17 aminopeptidases favoring large, non-polar residues, and each family also demonstrating quite unique intrapeptide specificities. Despite this, several studies have shown that the *P. falciparum* M1 and M17 aminopeptidases, and more recently, the *P. vivax* M1 and M17 aminopeptidases can be potently inhibited by a single compound with dual-target and cross-species activity [8–11]. The naturally occurring broad spectrum aminopeptidases inhibitor, bestatin, was used to investigate the potential for a single compound to inhibit *P. falciparum*, *P. vivax* and *P. berghei* M1 and M17 enzymes. Bestatin is only a moderate inhibitor of *Pf*A-M1 but showed inhibitory activity in the nanomolar range against the other five aminopeptidases (Table 3). *Pf*A-M1 was inhibited with the lowest potency (1.9 μM), whereas *Pf*A*-*M17 was inhibited with the highest potency (53 nM). The *P. vivax* and *P. berghei* enzymes all exhibited inhibitory constants between 100 and 350 nM. That both the M1 and M17 aminopeptidases from the three *Plasmodium* species could be inhibited with no distinct preference for either family is further evidence that cross-species and cross-peptidase family inhibitory targeting is a viable strategy in designing novel anti-malarial agents.

**Table 3:** Inhibition of M1 and M17 aminopeptidases by bestatin

	*k*_i_^(app)^ ± SEM (nM)					
	*Pf*A-M1	*Pv-*M1	*Pb*-M1	*Pf*A*-*M17	*Pv-*M17	*Pb*-M17
Bestatin	1880 ± 77	120 ± 6	117 ± 13	53 ± 8	347 ± 23	185 ± 20

*k*_i_^(app)^ calculated from three biological replicates and presented as mean ± SEM.

## Discussion

As drug resistance to frontline antimalarials continues to proliferate, there is an urgent need to generate new agents toward novel drug targets [21]. *Plasmodium* M1 and M17 aminopeptidases are validated targets for the design of novel anti-malarial agents, and the subject of ongoing structure-based drug design programs [8–11,22–25]. The development of potent and selective inhibitors requires a thorough characterization and comparison of how the target aminopeptidases function. Profiling kinetic fingerprints and identifying divergent versus conserved features provides insight into the inhibition of enzymes across families and species. Furthermore, by profiling the *P. berghei* enzymes in addition to *P. falciparum* and *P. vivax*, we are ensuring that structure-activity relationship data is applicable to relevant animal (mouse) trials. The work we have presented here sheds light on kinetic characteristics that are conserved within the M1 and M17 aminopeptidase families that can be exploited to achieve cross-species inhibition.

M1 aminopeptidases possess a broad P1 specificity profile and can generally be divided into two categories: favoring charged P1 residues (as seen with *Pb*-M1) [26,27], or favoring hydrophobic P1 residues (as seen with *Pf*A-M1 and *Pv*-M1) [26,28]. P1 specificity was generally conserved between the M1 homologs, although *Pf*A-M1 demonstrated a broader acceptance of residues than *Pv*-M1 and *Pb*-M1 did. *Pf*A-M1 also consistently demonstrated slower substrate turnover rates of both the Leu-Mec and hexapeptide substrates than *Pv*-M1 and *Pb*-M1. All residues involved in active site metal-coordination and architecture are conserved between *Pf*A-M1, *Pv*-M1 and *Pb*-M1, as are *K*_M_ values for the Leu-Mec substrate, suggesting that differences in catalytic efficiency are unlikely a result of differences in the hydrolysis mechanism. Discrepancies in catalytic efficiency and P1 specificity may possibly be attributed more to overarching structural and/or dynamic differences that cannot be differentiated between homologs from sequence alignment alone.

M1 family aminopeptidases undergo large-scale dynamic changes between an open conformation that exposes the internal active site, and a closed conformation [17,29,30]. When in the open conformation, the active site is disordered and the enzymes are inactive, suggesting that the enzyme must be in the closed conformation to restore catalytic activity [29,30]. Activation of the closing motion is postulated to occur either through an ‘induced fit’ mechanism, whereby peptide substrate and/or ligand binding in the active site induces a transition to the closed conformation [29,30]. Yeast LTA4H demonstrates this type of ‘induced fit’ mechanism as coordination of bestatin in the active site induces the closing motion and structural ordering of the active site [31]. A second mechanism has been proposed whereby two small peptides, or one long peptide, simultaneously occupy the active site and a structurally distinct allosteric site to initiate enzyme closing and substrate hydrolysis, as observed for the human M1 homolog ERAP1 [17,29,32]. Upon completion of the hydrolysis reaction, the opening motion mediates the release of the hydrolyzed products and recruitment of new substrates. The rate at which the enzyme can transition between these open and closed states thereby plays a role in the rate at which substrate and product are turned over. *Pf*A-M1 processed substrates at rates much lower than *Pv*-M1 and *Pb*-M1, suggesting that *Pf*A-M1 has a slower transition between open and closed conformations. A consequence of this slow transition may be extended exposure of the active site, which could allow for less favorable substrates to bind and result in the broad specificity profile that is only observed for *Pf*A-M1. Similarly, this slow movement may deter inhibitory compounds from binding tightly within the active site resulting in decreased compound potency compared with *Pv*-M1 and *Pb*-M1. Conversely, we postulate that the faster substrate turnover rates and more potent inhibition of *Pv*-M1 and *Pb*-M1 may correlate to an increase in dynamic behavior. The rapid transition between conformations appears to come at a specificity cost however, as both *Pv*-M1 and *Pb*-M1 exhibit narrower substrate specificity profiles than *Pf*A-M1. Analysis of the M1 aminopeptidase dynamics would be an incredibly interesting avenue to explore, as dynamic movement appears to be integral to enzyme function and warrants further investigation. Application of in-solution biophysical characterization, molecular dynamics, and mutation studies could help to identify the driving forces involved in the opening and closing mechanism. M1 aminopeptidases feature a conserved helix at the base of domain IV that is displaced when the enzyme is in the open conformation. Structural alignment of the three M1 homologs indicated that the loop connecting this helix to the following helix in both *Pv*-M1 and *Pb*-M1 diverged from *Pf*A-M1 (residues 789–801) in both sequence and length (Supplementary Figure S1). This loop would be an ideal candidate for structural mutation studies.

*Pf*A-M1 has been shown to have activity against longer peptides *in vivo*, digesting the hemoglobin-derived hexa-peptide, LDPENF [4]. This hexapeptide was digested by *Pf*A-M1, *Pv*-M1 and *Pb*-M1 *in vitro* and was therefore used as a template to design the alanine screen peptides to probe downstream substrate specificity. Throughout the alanine screen experiments, *Pv*-M1 and *Pb*-M1 continued to demonstrate the elevated turnover rates that were observed in earlier experiments. *Pv*-M1 in particular exhibited extremely fast turnover rates of the hexapeptides relative to *Pf*A-M1 and *Pb*-M1, perhaps indicating that *Pv*-M1 is more efficient at processing long substrates than the other two homologs. *Pv*-M1 was also the least affected by intrapeptide residue substitutions (with the exception of LDPENA), which is likely due to the very rapid turnover rate within the timeframe of the experiment rather than true intrapeptide specificity. The M1 aminopeptidases demonstrated a conserved preference for Leu over Ala in the P1 position, a result that is also supported by the P1 substrate screen. This P1 specificity is actually contradictory to the family classification as ‘alanyl aminopeptidases’ and is an example of misleading and confusing historic naming systems.

The three M1 homologs shared a conserved preference for peptide substrates LAPENF and LDPANF over LDPENF. The active site and substrate-binding cleft of *Pf*A-M1 is highly hydrophobic and therefore has a low tolerance for negatively charged residues such as Asp and Glu. These residues were also unfavorable in the P1 position, as none of the homologs successfully processed the Asp or Glu substrates (except for *Pf*A-M1, which shows minimal activity against Glu) (Figure 3). LDAENF digestion was a point of difference between *Pv*-M1/*Pb*-M1 and *Pf*A-M1. *Pv*-M1 and *Pb*-M1 favored Ala in the P2′ position over Pro, digesting LDAENF more efficiently than the template peptide LDPENF. Conversely, *Pf*A-M1 digested LDPENF more efficiently than LDAENF, indicating a preference for Pro at the P2′ position. We postulated that this may be due to structural differences in the S2′ binding sites of *Pv*-M1 and *Pb*-M1 compared with *Pf*A-M1 that would result in specificity towards Ala over Pro. However, the S2′ binding site of *Pf*A-M1 has not previously been mapped so direct comparison and prediction of the residues involved was not achievable. The *E. coli* APN crystal structure in complex with inhibitory molecule, PL250, has a defined S2′ site, and was superimposed onto the *Pf*A-M1 structure to estimate where the *Pf*A-M1 S2′ site is [15,33]. We estimated that the *Pf*A-M1 S2′ binding pocket is lined by residues Val459, Tyr575, Thr576 and Gln1038 (Supplementary Figure S6). These residues are conserved in *Pv*-M1 and *Pb*-M1, indicating that the S2′ site structure is also likely conserved. The difference in activity against LDAENF may therefore be a result of unique dynamicity between the homologs, with *Pv*-M1 and *Pb*-M1 accepting flexible peptides with a higher tolerance than *Pf*A-M1.

The *Plasmodium* M17 aminopeptidases were conserved in their preference for large and non-polar residues in the P1 position such as Leu, Tyr, Trp and Phe. The limited capacity for residues in the P1 position is primarily due to the hydrophobic nature of the active site, and although the crystal structure of *Pb*-M17 is not yet available, the high degree of sequence conservation in these regions likely means the hydrophobic nature is also conserved in *Pb*-M17. The *Plasmodium* M17 aminopeptidases have a highly limited specificity fingerprint when compared with homologs from porcine kidney (pkLAP), tomato (LAP-A), *E. coli* (PepA), *Helicobacter pylori* (*Hp*M17AP) and *Staphylococcus aureus* (*Sa*M17AP) which all show capacity to process a wider range of residues in the P1 position, such as Arg and Ala [18,34,35]. The ability to process substrates with Arg in the P1 position is associated with a more hydrophilic active site cavity, as seen with *Hp*M17AP and *Sa*M17AP, and the inclusion of a sodium ion at the base of the helix connecting the C- and N-terminal domains [34,35].

Activity levels against the alanine screen peptides compared with the hemoglobin-derived hexapeptide (LDPENF) varied between homologs, however the overall preference pattern remained conserved. *Pv-*M17 and *Pb*-M17 both had a low tolerance for Ala in the P4′ position in place of Asn, whereas *Pf*A*-*M17 tolerated Ala and Asn similarly in the P4′ position. The reason for this behavioral difference is unclear, as the location of the S4′ binding site within M17 aminopeptidases is unknown. Further to this, the known substrate sites (S1 and S1′) are highly conserved, as are the surrounding regions where the downstream binding pockets may be located. *Pv-*M17 and *Pb*-M17 preference for Asn in the P4′ position may be more related to substrate access to the active site through the N-terminal channels rather than substrate recognition and binding in the substrate binding sites. The three homologs shared a low tolerance for Ala in the P1, P2′ and P5′ position. The low activity against ADPENF was unsurprising, as the M17 aminopeptidases were shown to have low or no activity against Ala-ACC in the P1 screens (Figure 3). The three homologs demonstrated no activity against LDPENA within the time period of this experiment. This peptide substrate is hydrolyzable; we saw in earlier *Pv-*M17 and *Pb*-M17 time trials that the substrate can be cleaved, albeit at a very slow rate compared with the other screen peptides (Supplementary Figure S5). M17 aminopeptidases have highly hydrophobic and buried active sites, which may require a level of hydrophobicity at the peptide C-terminus to successfully interact with and bind the substrate-binding sites. Substitutions in the P1′ and P3′ positions generally had less of an influence on enzyme activity, possibly because these sites do not require specific residues, or because Ala also happens to be well-accepted in these positions.

Ongoing structure-based drug design studies have used the *P. falciparum* M1 and M17 X-ray crystal structures to design compounds that competitively occupy the S1 and S1′ subsites [8–11]. Cross-species and cross-family inhibition of *P. falciparum* and *P. vivax* M1 and M17 aminopeptidases has previously been demonstrated using these designed compounds . In this study, we used bestatin to show that cross-species inhibition extends across multiple *Plasmodium* species, and into *Plasmodium* species used in the animal model stages of drug development. We further demonstrated that the *P. falciparum*, *P. vivax* and *P. berghei* M1 and M17 families have conserved substrate preferences at the P1 position, and conserved responses when the P1 and P1′ residues of the hemoglobin-derived hexapeptide were substituted for alanine. This, together with the high sequence identity between homologs, indicates that inhibitory compounds designed using the *P. falciparum* active site structural information are likely to be highly effective against the *P. vivax* and *P. berghei* homologs. This comparative characterization information aids in the progression of compound design when X-ray crystal structures are not available, as is the case for *Pv*-M1, *Pb*-M1 and *Pb*-M17.

## Conclusions

In this study, we have shown that the M1 and M17 aminopeptidases from three *Plasmodium* species display largely conserved substrate specificity profiles and kinetic behaviors within their families. We showed that *Plasmodium* M1 and M17 aminopeptidases have distinct preferences for residues at various intra-peptide sequence positions, not just at the N-terminus. Further characterization of subsite specificities would provide invaluable insight into the functionality of M17, and M1, aminopeptidases and would be a welcome addition to the profiles outlined in this study. We can use this knowledge to broaden our understanding of the M1 and M17 aminopeptidase families and more specifically use our finding to anticipate challenges in designing inhibitory compounds that are engineered for both selective, cross-species and cross-family targeting.

## Data Availability

All supporting data, materials and sequence information are included within the main article and its Supplementary Files.

## Open Access Statement

Open access for this article was enabled by the participation of Monash University in an all-inclusive Read & Publish pilot with Portland Press and the Biochemical Society under a transformative agreement with CAUL.

## Abbreviations

ACC: 7-amino-4-carbamoylmethylcoumarin
LAP: Leucine Aminopeptidase
Leu-Mec: Leucine-7-amido-4-methyl-coumarin
NHMec: 7-amido-4-methyl-coumarin
