# Supplementary material for: Mapping the substrate specificity of the Plasmodium M1 and M17 aminopeptidases
Source: Biochem J. 2021 Jul 16;478(13):2697–713. doi: 10.1042/BCJ20210172 (PMC8286833; doi:10.1042/BCJ20210172)
Supplement: Supplementary Figures S1-S6 [file BCJ-478-2697-s1.pdf]

SUPPLEMENTARY MATERIAL

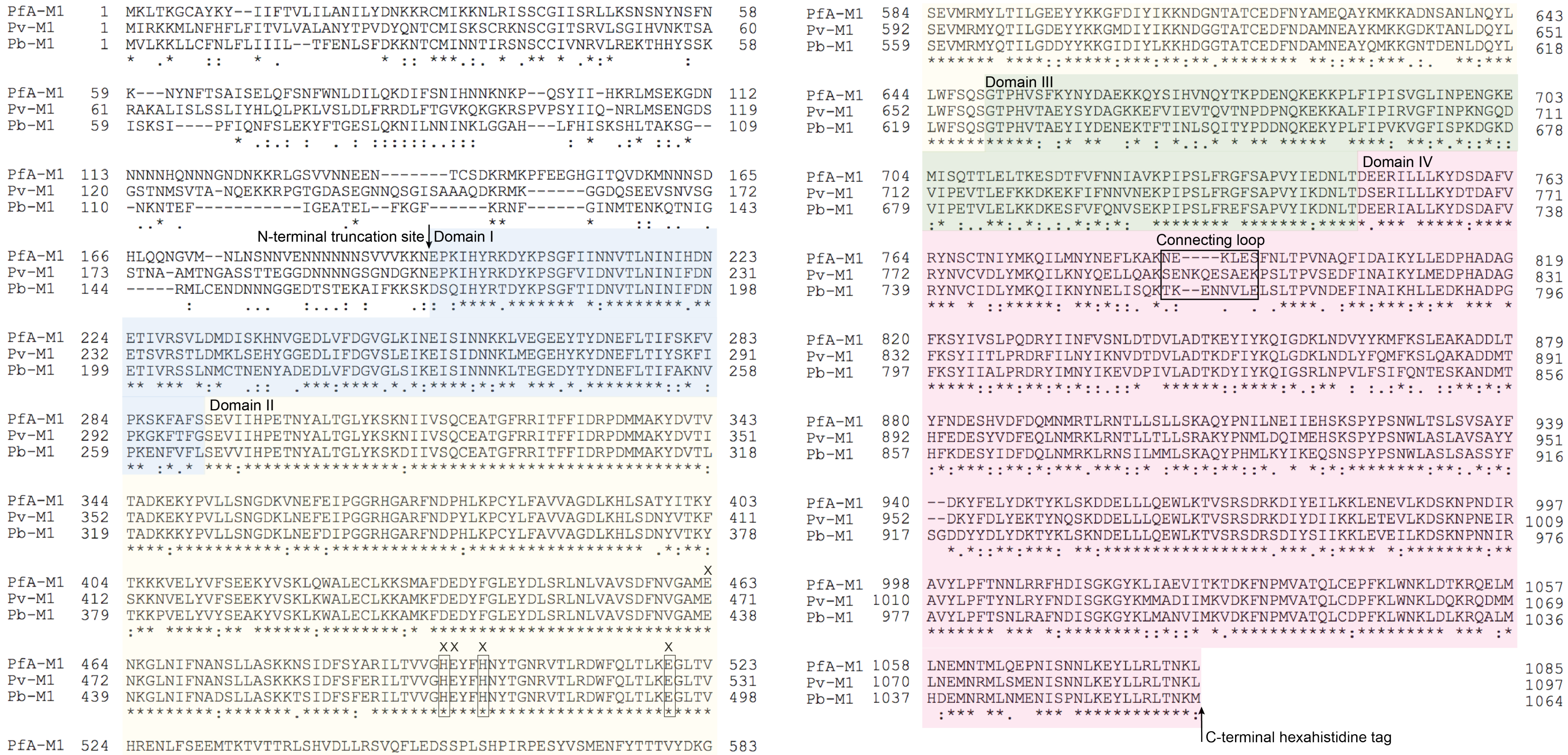

Supplementary Figure S1. Sequence alignment of *Pf*A-M1, *Pv*-M1 and *Pb*-M1, colored by domain.

Amino acid sequence alignment of *Pf*A-M1, *Pv*-M1 and *Pb*-M1. Domain I highlighted in blue, domain II in yellow, domain III in green and domain IV in pink. N-terminal truncation sites and C-terminal histidine site are labeled, conserved metal-binding residues are marked with ‘x’ and conserved active site residues boxed.

```
PfA-M17    1   MYFSSLCKFLPIS----EKEKIYLNIVKKRFC--------------------KSNIYYNN    36
Pv-M17     1   ---------MPLLRSSQHIKNTYWNIPKKSFR--------------------TGVPQFAE    31
Pb-M17     1   MYLTRLNINFV---DKIFENKFYKNIIKRYYFYNIKHSQKKFFFSFFISNSIKNKNNYNS    57
                         :          :: * ** *: :                   ..   : .
                                                   N-terminal truncation site ↓
PfA-M17   37   NNNNIINYNKRGLKFYP--------FCNNLKKNINFVNINNKKGINFHSINKERKMASEV    88
Pv-M17    32   ------SKKTRILHLHP--------LCKSASGVESPPFFDSQTFSSISNRKEFRKMATTV    77
Pb-M17    58   DCREFLTFNTYHYQKTPKYKIESLNFSKTIKKFYS---TNNIK-NFNISFSKFEKMSLKV   113
                     . :.   :  *          :.:. .   .     :. .     . .: .**:  *
               N-terminal domain
PfA-M17   89   PQVVSLDPTSIPIEYNTPIHDIKVQVYDIK-GGCNVEEGLTIFLVNNPGK----------   137
Pv-M17    78   PQVVSLDPTTIPIDYHTPIDDLSIEVKDISAEACPADEGLIVFLLNSAPKHSSSGGSGGN   137
Pb-M17   114   PQVINLDPTVLPVNYTTPIDDIEIVLKDGIKESCNFDDGLAIFLVNSKSEK---------   164
               ***:.**** :*::* ***.*:.: : *    .*  ::** :**:*.  :

PfA-M17  138   -----------ENGPVKISSKVNDKNVSEFLKDENMEKFNVKLGTSKHFYMFNDNKNSVA   186
Pv-M17   138   GGSAGSSGNGEGGAQIKINSSVKDNTINEFLKEGNMENFTGKLGTSKSFYIANDQKKYVS   197
Pb-M17   165   -----------DNGNTKISSQIKDSKINEFLSK-NDDIFNGKLGTFKSFYMANEKNKYIN   212
                          ..  **.*.::*..:.***.. * : *. **** * **: *:::: :

PfA-M17  187   VGYVGCGSVAD-LSEADMKRVVLSLVTMLHDNKLSKLTVVFEINVDKNLFRFFLETLFYE   245
Pv-M17   198   LAYVGCGPANE-ETELEIRKVAYALVTLLHDSKHKKVSIIFEIKIEEALFRFFLEHLFYE   256
Pb-M17   213   LSFIRCGTIDEEMTEFEIRKIVSSLVQILHDNKSVSTSIIFEIGINESLFRFFLETVFYE   272
               :.:: **   :  :* :::::. :** :***.*  . :::*** ::: ******* :***
                                                  Helical linker domain
PfA-M17  246   YMTDERFKSTDKNV--NMEYIKHLGVYINN-ADTYKEEVEKARVYYFGTYYASQLIAAPS   302
Pv-M17   257   YVTDERFKSADKST--ETDFIKNLSLHIAN-ADAYKGQIDKARVYFYGTYYAAQLIAAPS   313
Pb-M17   273   YVVDERFKSNDNKSSGNSENMKNLQIFLRNYNNNYNKQVKKSRTYFMGTYFASQLISAPS   332
               *:.****** *:.    : : :*:* :.: *   : *: ::.*:*.*: ***:*:***:***
               C-terminal domain
PfA-M17  303   NYCNPVSLSNAAVELAQKLNLEYKILGVKELEELKMGAYLSVGKGSMYPNKFIHLTYKSK   362
Pv-M17   314   NYCNPVSLSNAAVELAQKVNLECKILDVKELEELKMGAYLSVGKGSMYPNKFIHLTYKGA   373
Pb-M17   333   NYCNPVSLANVSVELAEKLNLECKILGIKELENLKMGAYLSVGKGSMYPNRFIHLTYKGK   392
               ********:*.:****:*:*** ***.:****:*****************:*******.
                              X    X      X             X
PfA-M17  363   -----GDVKKKIALVGKGITFDSGGYNLKAAPGSMIDLMKFDMSGCAAVLGCAYCVGTLK   417
Pv-M17   374   QTGASQNEKKKIALIGKGITFDSGGYNLKAAPGSMIDLMKFDMSGCAAVLGCAYCIGTIK   433
Pb-M17   393   -----GDIKKKIALVGKGITFDSGGYNLKASPGSMIDLMKFDMSGCAAVLGCAYCIGSIK   447
                    : ******:***********:***************************:*::*
                                                         X X
PfA-M17  418   PENVEIHFLSAVCENMVSKNSYRPGDIITASNGKTIEVGNTDAEGRLTLADALVYAEKLG   477
Pv-M17   434   PDNVEVHFLSAVCENMVSKNSYRPGDIITASNGKTIEVGNTDAEGRLTLADALVYAEKLG   493
Pb-M17   448   PENVEVHFLSAVCENMVSKNSYRPGDIITASNGKTIEVGNTDAEGRLTLADALVYAENIG   507
               *:***:*******************************************************::*

PfA-M17  478   VDYIVDIATLTGAMLYSLGTSYAGVFGNNEELINKILNSSKTSNEPVWWLPIINEYRATL   537
Pv-M17   494   VDYIVDIATLTGAMLYSLGTSYAGVFGNNDQLINKILSSSKTSNEPVWWLPIINEYRSSL   553
Pb-M17   508   VDHIIDIATLTGAMLYSLGTSYAGVFGNDNKLINKILESSKSSNEPVWWLPIIKEYRASL   567
               **:*:***********************:::******.***:***********:***::*

PfA-M17  538   NSKYADINNISSSVKASSIVASLFLKEFVQNTAWAHIDIAGVSWNFKARKPKGFGVRLLT   597
Pv-M17   554   NSKYADLNNISSSVKASSVVASLFLKEFIENTPWAHIDIAGVSWNFKARKPKGFGVRLLT   613
Pb-M17   568   NSKYADINNISSNVKASSVVASLFLNEFIQSTSWAHIDIAGVAWNFKDRKPKGFGVRLLS   627
               ******:*****.*****:******:**::.* *********:**** ***********:

PfA-M17  598   EFVLNDAL   605
Pv-M17   614   EFVLNDAV   621
Pb-M17   628   EFILNHSI   635
               **:**.::↑
                       C-terminal hexahistidine tag
```

Supplementary Figure S2. Sequence alignment of *Pf*A-M17, *Pv*-M17 and *Pb*-M17, colored by domain.
Amino acid sequence alignment of *Pf*A-M17, *Pv*-M17 and *Pb*-M17. Domain I highlighted in blue, domain II in yellow, and domain III in green. N-terminal truncation sites and C-terminal histidine site are labeled, conserved metal-binding residues are marked with ‘x’ and conserved active site residues boxed.

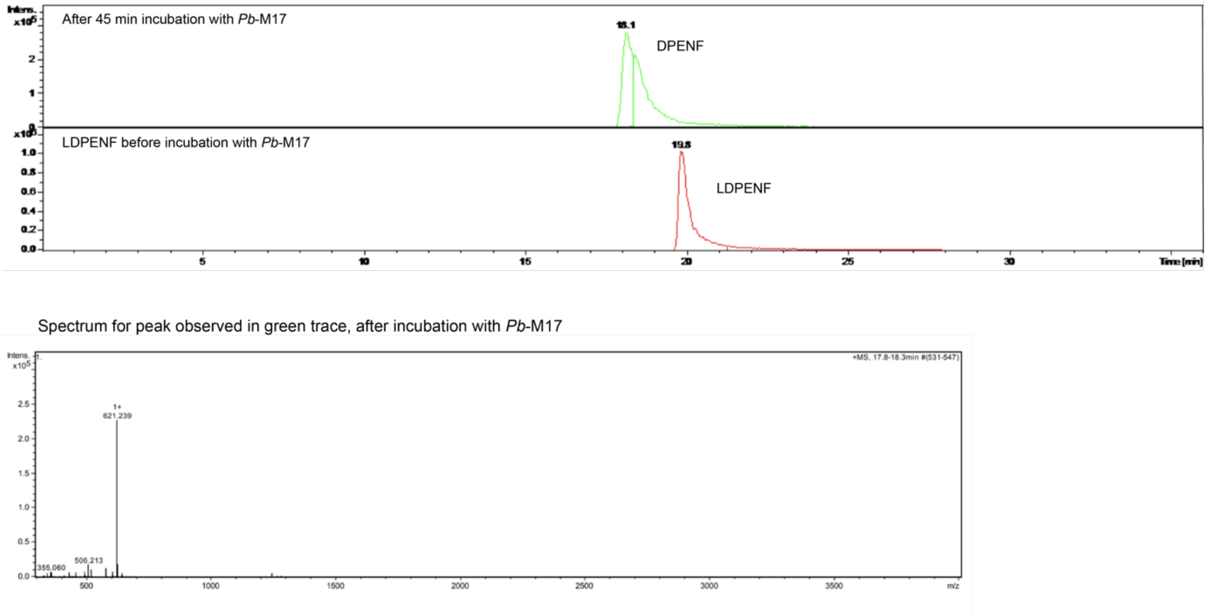

Supplementary Figure S3. Representative mass spectrometry trace of LDPENF digestion by *Pb*-M17. After 45 min of incubation, only one product at a molecular weight of 621 Da is observed. This correlated to the removal of the single amino acid residue Leu from the N-terminus of hexapeptide LDPENF, producing pentapeptide DPENF. Trace is representative of cleavage results obtained for each aminopeptidase – in each experiment only peptides of molecular weights 734 Da (LDPENF) and 621 Da (DPENF) were observed.

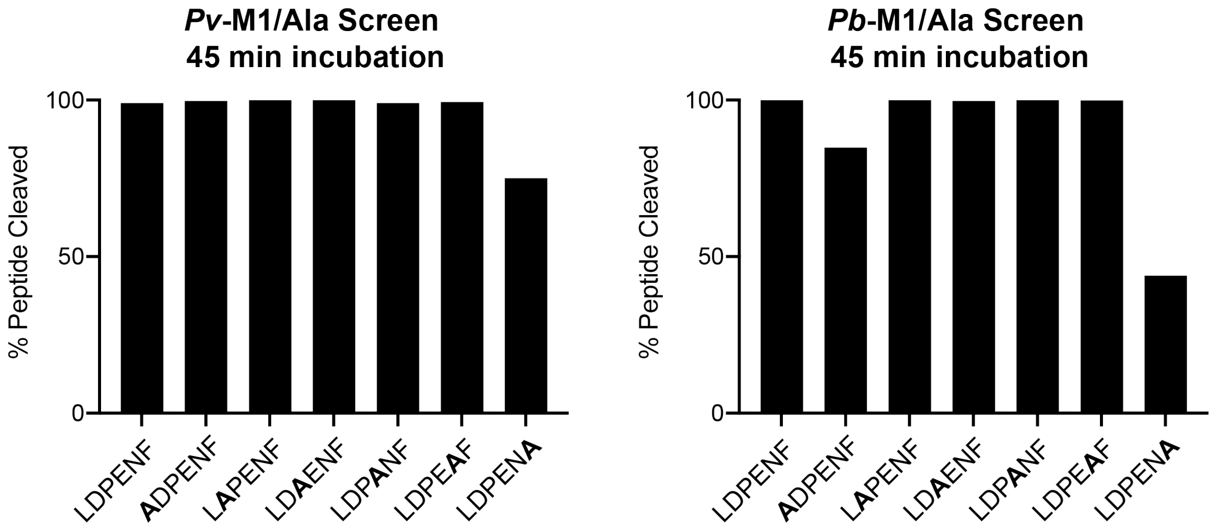

Supplementary Figure S4. Digestion of Ala screen peptides by *Pv*-M1 and *Pb*-M1 in 45-min incubation period. Template hexapeptide (LDPENF) and Ala screen peptides were digested to almost 100% in the 45-min incubation period at 37˚C. *Pv*-M1 and *Pb*-M1 showed decreased activity levels against peptides ADPENF and LDPENA.

.

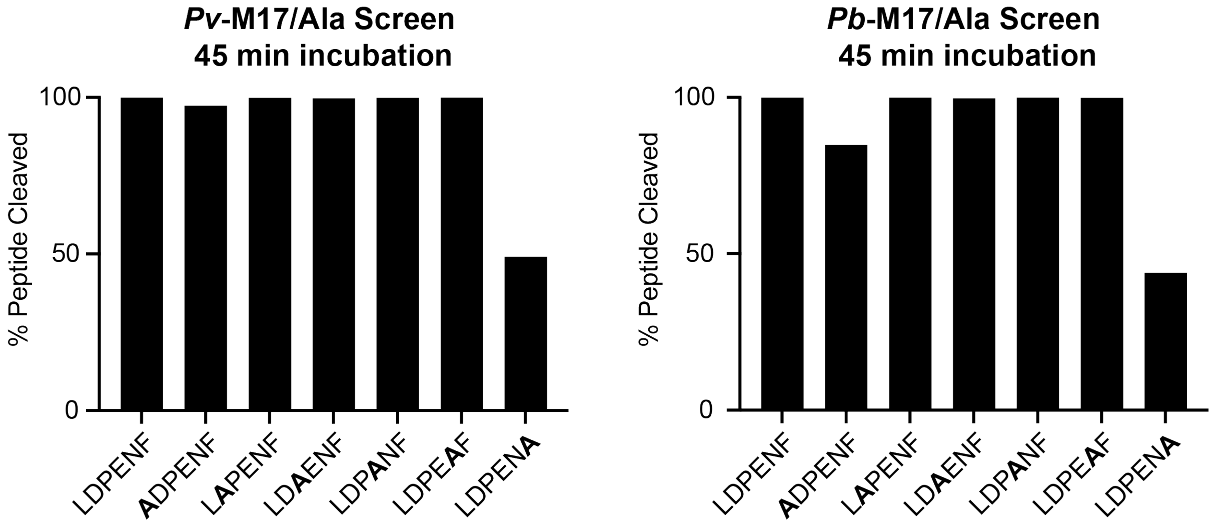

Supplementary Figure S5. Digestion of Ala screen peptides by *Pv*-M17 and *Pb*-M17 in 45-min incubation period. Template hexapeptide (LDPENF) and Ala screen peptides were digested to almost 100% in the 45-min incubation period at 37˚C. *Pv*-M17 and *Pb*-M17 showed decreased activity levels against peptides ADPENF and LDPENA compared to LDPENF.

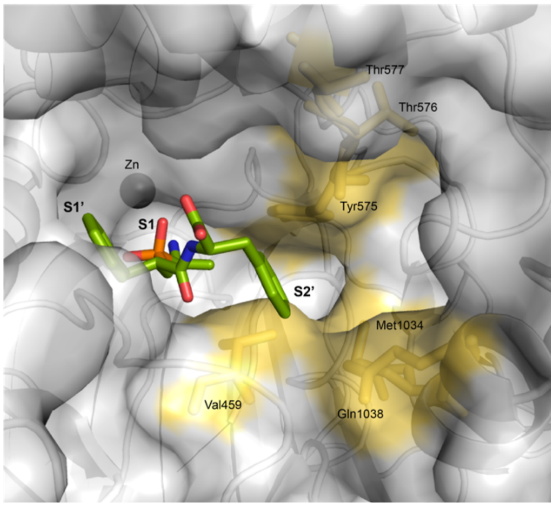

Supplementary Figure S6. APN inhibitor PL250 docked into *Pf*A-M1 X-ray crystal structure with P2' subsite highlighted.
E. coli APN X-ray crystal structure in complex with PL250 (PDB ID: 2ZXG) used to dock PL250 (green sticks) into *Pf*A-M1 X-ray crystal structure (PDB ID: 3EBG). S1, S1' and S2' pockets labelled. Residues postulated
